# Herpes simplex virus type 2 seroprevalence and associated factors in fertility-treatment-seeking population: A cross-sectional survey in the United Arab Emirates

**DOI:** 10.3389/fpubh.2022.991040

**Published:** 2022-11-07

**Authors:** Noor Motea Abdo, Irfan Aslam, Shazia Irfan, Junu A. George, Ahmed R. Alsuwaidi, Luai A. Ahmed, Rami H. Al-Rifai

**Affiliations:** ^1^Institute of Public Health, College of Medicine and Health Sciences, United Arab Emirates University, Al Ain, United Arab Emirates; ^2^HealthPlus Fertility and Genetics Center, Abu Dhabi, United Arab Emirates; ^3^Department of Pediatrics, College of Medicine and Health Sciences, United Arab Emirates University, Al Ain, United Arab Emirates; ^4^Zayed Center for Health Sciences, Al Ain, United Arab Emirates

**Keywords:** herpes simplex virus type 2 (HSV-2), reproductive health, infertility, fertility, pregnancy, genital infections

## Abstract

**Background:**

Herpes simplex virus type 2 (HSV-2) is a common genitally-transmitted viral infection affecting more than 400 million individuals globally. In the United Arab Emirates (UAE), in specific at-risk population groups, the burden of HSV-2 has not been reported. This study investigated the prevalence of HSV-2 IgG antibodies in patients seeking fertility treatment and characterized patients with seropositivity to HSV-2 IgG antibodies.

**Methodology:**

A cross-sectional sample of patients seeking fertility treatment in a major fertility clinic in Abu Dhabi, UAE was surveyed from April to May 2021. Patients were consecutively invited to complete self-administered questionnaires and provide blood for HSV-2 testing. Information on sociodemographics, medical history, and infertility was collected. Serum specimens were screened using an enzyme-linked immunosorbent assay for HSV-2 IgG antibodies detection.

**Results:**

Two hundred and ninety-nine patients were surveyed and provided blood samples. The mean age of the patients was 35.9 ± 6.8 [mean ± standard deviation (SD)] years with 89.3% being women. Sixty-six percent were overweight or obese, 25.0% had at least one chronic comorbidity, and 19.6% reported ever-had genital infection. More than two-thirds (68.3%) of the patients were infertile for ≥ 6 months. Of the 42 infertile males, 69.0% had an abnormal semen analysis. HSV-2 IgG antibodies was detected in 12.4% of patients. The HSV-2 IgG seropositive patients had a higher mean age (39.5 vs. 35.4 years; p < 0.001) compared to seronegative patients. HSV-2 IgG antibodies seropositivity was more common in males (15.6%) than females (12.0%), in patients with secondary (14.1%) vs. primary (9.2%) infertility, or in males with abnormal (10.3%) vs. normal (7.7%) semen.

**Conclusion:**

Exposure to HSV-2 at any time in patients seeking fertility treatment in the UAE was found to be slightly common in more than one out of 10 patients. Tailored health campaigns on HSV-2 prevention are warranted.

## Introduction

Herpes simplex viruses, in particular type 2 (HSV-2), which belong to the *Herpesviridae* family (1) is one of the prevalent genital infections affecting more than 400 million individuals worldwide (2). According to the World Health Organization (WHO), in 2016, the global prevalence of HSV-2 in people aged 15–49 years was 13% (3). HSV-2 is a contagious, lifelong, asymptomatic infection during the latent stage. When reactivated, clinical manifestations are characterized by itchy blisters and painful sores in the genital area that lasts 2–4 weeks (1). Symptoms can be severe during the initial episode of infection, and for immunosuppressed patients, lead to severe complications. HSV-2 is responsible for 99% of HSV-related genital ulcer diseases (GUD). Globally, in 2016, around 178 million persons aged 15–49 years suffered from GUD (4). In addition, HSV-2 infection is associated with a 2–4-fold increase in the risk of acquiring human immunodeficiency virus (HIV) even when treated (5). At the time of labor, mother-to-neonates transmission of HSV infection was reported in asymptomatic mothers with subclinical HSV infection (6, 7). Infection with HSV in neonates was associated with premature delivery and low birth weight babies (6). Neonatal death in which neonatal mortality rate due to HSV-2 is estimated at 80% but reduced to 29% after treatment with antiviral therapy (8).

Age, sex, and ethnicity were described to have a role in acquiring HSV-2 (1). In the United States, during the period 2015–2016 among people in their reproductive age period, females were reported to be almost two times more likely to contract HSV-2 compared to males (9). Ethnicity also contributes to the spread of HSV-2 infection. The prevalence of HSV-2 was reported to be higher in non-Hispanic blacks when compared with non-Hispanic whites (10). Advanced age was also associated with HSV-2 infection (3).

A major concern of HSV-2 is infertility and infertility-related complications, including pelvic inflammatory disease, ectopic pregnancy, and recurrent miscarriage (11). Genital HSV-2 was reported to be associated with abortion, intrauterine growth retardation (12) and abnormal semen (13). The effect of HSV-2 on male infertility has been well-investigated. Studies have shown that HSV-2 infection is associated with abnormal semen parameters (13).

In the United Arab Emirates (UAE), one in every six couples have reported difficulties in conceiving babies (14). The fertility rate in the UAE has decreased from 2.6 per 100,000 in 2009 to 1.4 per 100,000 in 2019 (15). It was estimated that 100,446 men and women face infertility problems in the UAE, and 50,223 cases of them are women. This estimate is higher than that recorded in different neighboring countries, such as Kuwait with 61,108 cases of infertility, Oman with 62,392 cases, and Qatar with 28,691 cases. In Dubai alone, the incidence of infertile women seeking treatment per year estimated to nearly double from 5,975 cases in 2015 to 9,139 cases by 2030. However, high-quality seroprevalence data and evidence on the burden of and factors associated with several genital pathogens in high-risk populations, such as infertile people in the UAE, are lacking.

According to general guidelines, population screening for HSV-2 is not recommended and limited to those who have history or at risk of infection (9). Possible transmission of HSV-2 infection may occur when genital symptoms are misdiagnosed. Therefore, the prevalence of HSV-2 is likely to be underestimated, and the real burden estimate is incomplete. Nowadays, HSV-2 type specific glycoprotein G-based enzyme-linked immunosorbent assays (ELISAs) are considered reliable tests to be used for population serological screening for research purposes. Investigating history of exposure to genital HSV-2 in specific populations, including infertile populations, is of paramount importance. The focus of this study was to investigate the history of exposure to and factors associated with HSV-2 infection in patients seeking fertility treatment in the Emirate of Abu Dhabi, UAE.

## Methods

### Study design, setting, and study population

The present cross-sectional study was conducted in a major fertility clinic in the Emirate of Abu Dhabi, UAE. During the period from April to May 2021, patients seeking fertility treatment were consecutively invited to participate in a self-administered questionnaire and to provide blood samples for serological investigation. Eligibility to participate was defined as any patient aged ≥18 years and seeking fertility treatment. Patients aged < 18 years, spontaneously pregnant women, and/or seeking healthcare other than for fertility-related health issues were excluded.

### Survey data collection

Well-trained interviewers clarified the study objectives and procedures and invited patients to participate in the study. Eligible participants consented to participate and completed a validated self-administered questionnaire. The questionnaire collected information on sociodemographic characteristics, including age, gender, nationality, education level, and marriage duration. Also, the questionnaire collected information on medical conditions, including body mass index (BMI), chronic comorbidities, history of genital infections, and fertility-related information. Additional necessary data were retrieved from medical files.

### Blood samples collection and screening for HSV-2 IgG antibodies

Trained and certified nurses collected 10 ml of whole blood in plain tubes. Blood samples were maintained under appropriate conditions in a refrigerator at 8 °C for further serum separation on the same collection day. The serum was separated and stored at −20°C for further serological testing. Sera were screened for the detection of HSV-2 immunoglobulin IgG class-specific glycoprotein gG2 using a commercially available ELISA kit (Euroimmun, Lübeck, Germany). All ELISA tests were performed by an expert laboratory technician following the manufacturer's instructions. According to the manufacturer, the selected ELISA kits have 100% sensitivity and 100% specificity for detecting HSV-2 IgG antibodies with no cross-reactivity with HSV-1. In each testing run, calibrators and positive and negative controls were used. Additionally, to ensure a quality testing procedure, 15% of the tested sera samples were randomly selected and tested in duplicate wells in the same run. HSV-2 IgG antibodies titer was interpreted according to the manufacturer's recommendation as either positive (titer ≥ 22 RU/ml), borderline (titer ≥ 16 to < 22 RU/ml), or negative (titer < 16 RU/ml).

### Sample size

Referring to the recently estimated global seroprevalence of HSV-2 in the infertile population of 12% (3) and using the sample size calculation formula for cross-sectional surveys, considering a power of 80% and significance level of ≤ 0.05, the minimum sample size was estimated at 210 subjects. After adjusting for a potential 35% blood-collection refusal rate, the minimum required sample size was increased to 300 subjects.

### Data management and statistical analysis

In addition to being described as continuous variables, age and BMI at the time of the survey were also described as categorical variables. Age was divided into three groups: (1) 19–30, (2) 31–40, and (3) 41–54 years and BMI into four groups: (1) underweight: < 18.5 kg/m^2^, (2) normal: 18.5–24.9 kg/m^2^, (3) overweight: 25.0–29.9 kg/m^2^, and (4) obese: ≥30.0 kg/m^2^. BMI was calculated based on body weight in kilograms (kg) divided by the squared height in meters (m^2^). Seropositivity to HSV-2 IgG antibodies was reported as either positive or negative. Sera samples (*n* = 8) with borderline for HSV-2 IgG antibodies titer were treated as negative to HSV-2 IgG antibodies. Other collected sociodemographic, medical, and fertility-related characteristics were reported as categorical variables.

Frequencies and valid percentages described categorical variables. A chi-square test was used to compare the HSV-2 IgG antibodies seropositive and seronegative patients against their measured categorical characteristics. A Shapiro–Wilk test was used to assess the normality of continuous variables. Median and interquartile range (IQR) of continuous variables that violated normality assumption were compared using non-parametric Mann–Whitney U tests for independent samples. Normal-distributed continuous variables were reported as mean ± standard deviation (SD) and compared by the two-sample *t*-test. The crude association between the measured characteristics and seropositivity to HSV-2 IgG antibodies was determined using logistic regression models to estimate the likelihood [odds ratio (OR)] of seropositivity to HSV-2 IgG antibodies by the function of the measured exposures.

The IBM SPSS Statistics version 26.0 was used for all statistical analyses with two-tailed *p*-values ≤ 0.05 defined as statistically significant.

### Ethics approval

The study protocol was approved by the Abu Dhabi Health Research and Technology Committee Institutional Review Board (Ref: DOH/CVDC/2020/1191). The study was also approved by the HealthPlus Research Ethics Committee (REC/2020/P13). The study was conducted in accordance with the Declaration of Helsinki and adhered to Good Clinical Practice guidelines. Consent forms were collected from each patient. Confidentiality was preserved by de-identifying all data.

## Results

### Patients' characteristics

During the study period, 299 patients were interviewed, their blood samples were obtained, and tested for HSV-2 IgG antibodies. The mean age of the patients was 35.9 years ± 6.8, and 89.3% were females or from a Middle Eastern country (91.2%). The mean BMI was 28.0 kg/m^2^ ± 5.4 with two-thirds (66.0%) of the patients overweight or obese. Most (93.2%) of the patients seeking fertility treatment were married for ≥ 1 year and nearly two-thirds (62.0%) for ≥ 5 years. Of the patients who reported their chronic comorbidities, a quarter had at least one chronic comorbidity with diabetes mellitus being the most common one (7.8%). Self-reported urinary tract and genital infections in the past 3 months were reported by 15.3 and 19.6%, respectively, with 45.5% of the patients reporting having had genital infections more than once (Table 1).

**Table 1:** Prevalence of HSV-2 IgG antibodies according to the sociodemographic and medical characteristics of the fertility clinics' attendees and the crude association with being seropositive.

	**All** ***N =* 299**	**HSV-2 IgG**		
	***n* (valid %)**	**Positive** ***n =* 37, (12.4%)**	**Negative** ***n =* 262, (87.6%)**	***P*–value**
		***n* (valid %)**	***n* (valid %)**	
**Age, years (mean ±SD)**	35.9 ± 6.8	39.5 ± 5.8	35.4 ± 6.8	< 0.001
Median (IQR)	36.0 (31–41)	41.0 (36.0–44.0)	35.0 (31.0–41.0)	< 0.001
19–30	68 (22.8)	4 (5.9)	64 (94.1)	0.007
31–40	141 (47.3)	14 (9.9)	127 (90.1)	
41–54	89 (29.9)	19 (21.3)	70 (78.7)	
Missing	1	0	1	
**Sex**				0.555
Male	32 (10.7)	5 (15.6)	27 (84.4)	
Female	267 (89.3)	32 (12.0)	235 (88.0)	
**Nationality**				
Middle East	269 (91.2)	35 (13.0)	234 (87.0)	0.220
Others	26 (8.8)	2 (5.4%)	24 (92.3)	0.434
Africa	7 (2.4)	2	5	
America, Asia, and Europe	19 (6.5)	0	19	
Missing	4	0	4	
**Education**				0.191
Secondary and below^a^	75 (26.0)	12 (16.0)	63 (84.0)	
College level and higher	213 (74.0)	22 (10.3)	191 (89.7)	
Missing	11	3	8	
**BMI kg/m**^**2**^ **(mean ±SD)**	28.0 ± 5.4	28.8 ± 4.6	27.9 ± 5.5	
Median (IQR)	27.4 (23.7–31.6)	28.3 (25.2–32.8)	27.2 (23.6–31.6)	0.545
Normal	97 (33.0)	9 (9.3)	88 (90.7)	0.368
Overweight	98 (33.3)	15 (15.3)	83 (84.7)	
Obese	96 (32.7)	13 (13.5)	83 (86.5)	
Underweight	3 (1.0)	0	3	
Overweight and obese	194 (66.0)	28 (14.4)	166 (85.6)	0.368
Missing	5	0	5	
**Marriage duration**				0.916
Less than a year	20 (6.8)	3 (15.0)	17 (85.0)	
1–5 years	92 (31.2)	12 (13.0)	80 (87.0)	
More than 5 years	183 (62.0)	22 (12.0)	161 (88.0)	
Missing	4	0	4	
**Chronic co–morbidity**				0.225
No	201 (75.0)	26 (12.9)	175 (87.1)	
At least one	67 (25.0)	5 (7.5)	62 (92.5)	
Missing	31	6	25	
Diabetes Mellitus^b^	21 (7.8)	4 (19.0)	17 (81.0)	0.264
Respiratory diseases^d^	17 (6.3)	0	17	0.123
Cardiovascular disorders^c^	10 (3.7)	0	10	0.244
Rheumatoid arthritis	1 (0.4)	0	1	0.717
Thyroid disorders^e^	9 (3.4)	1	8	0.965
Other chronic diseases^f^	16 (6.0)	1	15	0.493
**Ever had urinary tract infection**				0.355
No	227 (84.7)	28 (12.3)	199 (87.7)	
Yes	41 (15.3)	3 (7.3)	38 (92.7)	
Missing	31	6	25	
**Ever had genital infection**				0.383
No	238 (80.4)	27 (11.3)	211 (88.7)	
Yes	58 (19.6)	9 (15.5)	49 (84.5)	
Missing	3	1	2	
**Frequency of genital infection**				0.780
Once	30 (54.5)	4 (13.3)	26 (86.7)	
More than once	25 (45.5)	4 (16.0)	21 (84.0)	
Missing	3	1	2	
**Genital infection symptoms occurrence^g^**				0.637
No	154 (59.2)	16 (10.4)	138 (89.6)	
At least one	106 (40.8)	13 (12.3)	93 (87.7)	
Missing	39	8	32	

a Five of were with no schooling.

b Diabetes Mellitus of any type including gestational diabetes mellitus.

c Cardiovascular disorders of any type including high blood pressure.

d Chronic respiratory diseases include chronic obstructive pulmonary disease, asthma and tuberculosis.

e Thyroid disorders of any type.

f Other chronic diseases include cancer, allergy, anemia, polycystic ovary syndrome and premenstrual dysphoric disorder.

g Currently or during the past 3 months.

IQR, Interquartile range; SD, Standard deviation.

Table 2 shows fertility-related characteristics. History of pregnancy loss or ectopic pregnancy was reported by 35.3 and 10.0%, respectively. More than two-thirds (68.3%) were infertile for ≥ 6 months with nearly 57.0% infertile for more than 12 months. The most common documented cause of infertility was female infertility of unspecified origin (87.5%). Of the infertile males, 69.0% had abnormal semen (Table 2).

**Table 2:** Prevalence of HSV-2 IgG antibodies according to the fertility characteristics of the fertility clinics' attendees and the crude association with being seropositive.

	**All** ***N =* 299**	**HSV-2 IgG**		
	***n* (valid %)**	**Positive** ***n =* 37, (12.4%)**	**Negative** ***n =* 262, (87.6%)**	***P*–value**
		***n* (valid %)**	***n* (valid %)**	
**Type of infertility**				0.218
Primary	109 (37.1)	10 (9.2)	99 (90.8)	
Secondary	185 (62.9)	26 (14.1)	159 (85.9)	
Missing	5	1	4	
**History of pregnancy loss**				0.318
No	187 (64.7)	20 (10.7)	167 (89.3)	
Yes	102 (35.3)	15 (14.7)	87 (85.3)	
Yes, once	52 (18.0)	8 (15.4)	44 (84.6)	0.594
Yes, recurrent loss	50 (17.3)	7 (14.0)	43 (86.0)	
Missing	10	2	8	
**History of ectopic pregnancy**				0.759
No	260 (90.0)	32 (12.3)	228 (87.7)	
Yes	29 (10.0)	3 (10.3)	26 (89.7)	
Yes, once	26 (9.0)	2 (7.7)	24 (92.3)	0.416
Yes, recurrent	3 (1.0)	1 (33.3)	2 (66.7)	
Missing	10	2	8	
**Infertility duration**				0.499
< 3 months	51 (17.6)	5 (9.8)	46 (90.2)	
3–6 months	41 (14.1)	7 (17.1)	34 (82.9)	
6–12 months	33 (11.4)	5 (15.2)	28 (84.8)	
>12 months	165 (56.9)	16 (9.7)	149 (90.3)	
≥6 months	198 (68.3)	21 (10.6)	177 (89.4)	0.458
Missing	9	4	5	
**Infertility causes^a^**				0.694
Female infertility of other or unspecified origin	237 (87.5)	28 (11.8)	209 (88.2)	
Male infertility/Other or unspecified	26 (9.6)	4 (15.4)	22 (84.6)	
Female infertility associated with anovulation	3 (1.1)	0	3	
Female infertility of tubal origin	2 (0.7)	0	2	
Female infertility of uterine origin	3 (1.1)	1 (33.3)	2 (66.7)	
Missing	28	4	24	
**Semen analysis^b^**				0.787
Normal	13 (31.0)	1 (7.7)	12 (92.3)	
Abnormal	29 (69.0)	3 (10.3)	26 (89.7)	
Azoospermia	8 (27.6)	2 (25.0)	6 (75.0)	0.199
Asthenospermia	1 (3.4)	0	1 (100.0)	
AsthenoTeratozospermia	6 (20.7)	0	6 (100.0)	
Oligospermia	2 (6.9)	0	2 (100.0)	
OligoAsthenospermia	1 (3.4)	0	1 (100.0)	
OligoTeratoAsthenospermia	2 (6.9)	0	2 (100.0)	
Teratospermia	9 (31.0)	1 (11.1)	8 (88.9)	

a According to ICD codes.

b Reported in medical records. In addition to the 32 surveyed males, husbands of surveyed females with available information in medical records on their semen analysis were added. Their wives screened for HSV-2 were considered as a proxy for HSV-2 IgG seropositivity in their husbands.

IQR, Interquartile range; SD, Standard deviation.

### Seroprevalence of HSV-2 IgG antibodies

Of the 299 tested sera samples, 12.4% were positive to HSV-2 IgG antibodies. Tables 1, 2 show the distribution and correlation between the measured characteristics and seropositivity to HSV-2 IgG antibodies. When compared with patients who tested seronegative, age was the only characteristic significantly associated with a difference in seropositivity to HSV-2 IgG antibodies. Patients who tested seropositive to HSV-2 IgG antibodies were older (mean age: 39.5 vs. 35.4 years, p < 0.001). In males, there were more seropositive to HSV-2 IgG antibodies compared to females (15.6 vs. 12.0%). Patients who reported having genital infections at any time had higher HSV-2 IgG antibodies seroprevalence values when compared with patients who did not (15.5 vs. 11.3%), particularly patients who had experienced genital infection more than once (16.0% vs. 13.3%) in the past 3 months as shown in Table 1.

Seroprevalence of HSV-2 IgG antibodies was 1.53-time higher in patients with secondary (14.1%) when compared with patients with primary (9.2%) infertility and 1.38-time higher in patients with a history of pregnancy loss (14.7%) when compared with other patients (10.7%). Regarding the duration of infertility, the highest HSV-2 IgG antibodies seropositivity was observed in patients who have been infertile for 3–6 months (17.1%) followed by 6 to 12 months (15.2%). Infertile males with abnormal semen had a higher (10.3%) prevalence of HSV-2 IgG antibodies compared to males with normal semen (7.7%) as shown in Tables 2, 3.

**Table 3:** The crude and adjusted association between the measured characteristics of fertility clinics' attendees with being of seropositive to HSV-2 IgG antibodies.

	**Crude OR**	**Age-adjusted OR**
**Age—years, continuous**	1.1 (1.0–1.2)^**^	–
19–30	1.00	–
31–40	1.8 (0.6–5.6)	–
41–54	4.3 (1.4–13.4)^*^	–
**Sex**		
Male	1.00	1.00
Female	0.7 (0.3–2.0)	0.8 (0.3–2.3)
**Nationality**		
Middle East	1.00	1.00
Others	0.6 (0.1–2.5)	0.5 (0.1- 2.3)
Africa	2.7 (0.5–14.3)	2.4 (0.4–13.7)
America, Asia, and Europe	NA	NA
**Education**		
Secondary and below^a^	1.00	1.00
College level and higher	0.6 (0.3–1.3)	0.6 (0.3–1.3)
**BMI** kg/m^2^ continuous	1.0 (0.9–1.1)	1.0 (0.9–1.1)
Normal	1.00	1.00
Overweight	1.8 (0.7–4.3)	1.4 (0.6–3.5)
Obese	1.5 (0.6–3.8)	1.1 (0.4–2.9)
Underweight	NA	NA
Overweight and obese	1.6 (0.7–3.6)	1.3 (0.6–2.9)
**Marriage duration**		
Less than a year	1.00	1.00
1–5 years	0.9 (0.2–3.3)	0.9 (0.2–3.6)
More than 5 years	0.8 (0.2–2.9)	0.5 (0.1–2.1)
**Chronic co–morbidity**		
No	1.00	1.00
At least one	0.5 (0.2–1.5)	0.5 (0.2–1.3)
Diabetes Mellitus^b^	1.9 (0.6–6.1)	1.3 (0.4–4.4)
Cardiovascular disorders^c^	–	
Respiratory diseases^d^	–	
Rheumatic arthritis	–	
Thyroid dysfunction^e^	1.0 (0.1–7.9)	0.7 (0.1–5.6)
Other chronic diseases^f^	0.5 (0.1–3.9)	0.5 (0.1–4.5)
**Ever had urinary tract infection**		
No	1.00	1.00
Yes	0.6 (0.2–1.9)	0.5 (0.2–1.9)
**Ever had genital infection**		
No	1.00	1.00
Yes	1.4 (0.6–3.2)	1.5 (0.6–3.3)
**Frequency of genital infection**		
Once	1.00	1.00
More than once	1.2 (0.3–5.6)	1.4 (0.3–6.5)
**Genital infection symptoms occurrence^g^**		
No	1.00	1.00
At least one	1.2 (0.6–2.6)	1.2 (0.6–2.7)
**History of pregnancy loss^h^**		
No	1.00	1.00
Yes	1.4 (0.7–3.0)	1.2 (0.6–2.5)
Yes, once	1.5 (0.6–3.7)	1.5 (0.6–3.6)
Yes, recurrent loss	1.4 (0.5–3.4)	1.0 (0.4–2.6)
**History of ectopic pregnancy^h^**		
No	1.00	1.00
Yes	0.8 (0.2–2.9)	0.8 (0.2–2.7)
Yes, once	0.6 (0.1–2.6)	0.6 (0.1–2.5)
Yes, recurrent	3.6 (0.3–40.4)	2.5 (0.2–28.9)
**Type of infertility**		
Primary	1.00	1.00
Secondary	1.6 (0.7–3.5)	1.3 (0.6–2.8)
**Infertility duration**		
< 3 months	1.00	1.00
3–6 months	1.9 (0.6–6.5)	2.1 (0.6–7.5)
6–12 months	1.6 (0.4–6.2)	1.7 (0.4–6.7)
>12 months	1.0 (0.3–2.8)	0.7 (0.2–2.2)
≥6 months	1.1 (0.4–3.1)	0.9 (0.3–2.5)
**Infertility causes^i^**		
Male infertility/Other or unspecified	1.00	1.00
Female infertility of other or unspecified origin	0.7 (0.2–2.3)	0.8 (0.2–2.4)
Female infertility associated with anovulation	–	–
Female infertility of tubal origin	–	–
Female infertility of uterine origin	2.8 (0.2–38.0)	4.2 (0.3–64.6)
**Semen analysis^j^**		
Normal	1.00	1.00
Abnormal	1.4 (0.1–14.7)	2.1 (0.2–23.7)

a Five patients reported no schooling.

b Diabetes Mellitus of any type including GDM.

c Cardiovascular disorders of any type including high blood pressure.

d Chronic respiratory diseases include OCPD, asthma and TB.

e Thyroid dysfunctions of any type.

f Other chronic diseases include cancer, allergy, anemia, PCOS, PMDD.

g Currently or during the past 3 months,

h Sample size includes females only (N = 255),

i According to ICD codes.

j Reported in medical records.

* p < 0.05,

** p < 0.001.

Table 3 shows the association between seropositivity to HSV-2 IgG antibodies and the measured characteristics. Only patients who were 41–54 years showed a significant association with a higher prevalence of HSV-2 IgG antibodies compared to patients 19–30 years old [odds ratio (OR): 4.3, 95% confidence interval (CI): 1.4–13.4; *p* = 0.011].

## Discussion

The present study reveals the burden of previous exposure to HSV-2 in patients seeking fertility treatment in the Abu Dhabi Emirate. The study revealed that at least one in ten patients have ever been exposed to HSV-2 by testing seropositive to HSV-2 IgG antibodies. The study also explores the association of several risk factors with seropositivity to HSV-2 IgG antibodies. Although seropositivity to HSV-2 IgG antibodies was found to vary across the measured characteristics, age was the only characteristic that was significantly associated with a higher prevalence of HSV-2 IgG antibodies. The present study used the HSV-2-specific glycoprotein G2 ELISA method for the detection of IgG-class antibodies in serum samples. This method has been extensively studied and evaluated in the literature and is considered a reliable method for HSV-2 screening in several populations (16). The perfect 100% sensitivity and specificity of the selected ELISA kits support this reliability.

The observed HSV-2 IgG antibodies prevalence of 12.4% in patients seeking infertility treatment is approximately two-fold higher than the prevalence of HSV-2 reported in females (6.5%) with symptomatic genital infections that have been reported in the UAE (17). However, this difference in the reported prevalence in the present study and the other study from UAE in 2010 (17) can be explained by the differences in the testing method. The present study uses seroprevalence based on HSV-2 IgG antibody testing, while the 6.5% HSV-2 prevalence is molecular-based. Our results were based on IgG antibodies, which capture previous exposure to HSV-2. The observed HSV-2 seroprevalence of 12.4% in patients seeking infertility treatment is 9.5-times higher than that reported in the general population in Saudi Arabia (18) and 3.6-times higher than that reported in Shandong Province in China (3.4%) (19). This higher any-time exposure to HSV-2 in patients seeking fertility treatment, particularly infertile males, compared to general and other population groups explains the reported association between infertility and HSV-2, particularly in males (13). However, the literature presents opposing results concerning the effects of HSV-2 on female infertility. Some experts simplify the role of HSV-2 on women as abstention from sexual intercourse, (19–21), yet multiple studies disclosed the potential impact of HSV-2 on female infertility. A study among infertile women in Rwanda revealed a significant association between seropositive HSV-2 and tubal infertility (21). Another study in Hungary documented higher HSV-2 prevalence among infertile compared to fertile women (22). In India, HSV-2 was associated with primary infertility in young women (23). In the present study, higher HSV-2 prevalence was observed among patients with secondary compared to those with primary infertility. The documented HSV-2 and male infertility can be explained by that fact that male genital infections with HSV-2 lead to inflammatory responses that affect not only semen characteristics but also cause testicular and other male reproductive organs damage leading to infertility (13, 24). In our study, higher HSV-2 IgG antibody levels were observed in males with abnormal semen. Few studies have proposed an association between HSV-2 and lower semen count (24). In Brazil, positive HSV-2 semen of infertile men was significantly associated with lower seminal volume and hemato-sperm (25). Although our results were not clinically significant, they were relevant, and more studies focusing on males with a proper sample size are advisable. In our population, patients suffering from pregnancy loss were more affected by HSV-2 infection. The literature is inconsistent in terms of reporting an association between pregnancy loss and HSV-2. A recently published review discussed the possible role of HSV-2 in miscarriage (26). A conclusion about this conflict could be crucial to further aid patients going through fertility treatment to avoid any possible pregnancy loss.

HSV-2 is a genital infection transmitted through genital contact for which it is vital to consider the socio-cultural nature of the UAE when interpreting the results of this study. Nearly 88% of the UAE population are expatriates coming from various backgrounds and behavioral practices (27). The observed higher prevalence of HSV-2 infection among males compared to females is possibly due to the potential for more risky behavior in men. Another interesting finding was the significant association between age and exposure at any time to HSV-2. This observation is in line with what is reported in the literature in Jordan (28), and the general population in Poland (29). As expected, a higher spread of HSV-2 infection among less-educated populations indicates the need for education and awareness programs. Stoner et al. reported an association between level of literacy and infection with HSV-2 (30). In terms of infertility, association with genital infections is well studied for different pathogens (31).

A few limitations should be considered when interpreting the present findings. The nature of the cross-sectional study hinders the establishment of a causal pathway with the measured characteristics and with the potential role of HSV-2 in infertility. Also, this study was limited to recruiting patients from only one fertility clinic. This restricts generalizability to general and specific populations and other fertility-treatment-seeking patients in the UAE. Moreover, although the manufacturer states a diagnostic sensitivity and specificity of 100%, cross-reactivity with HSV-1 is possible due to the antigenic similarity between these viruses. Furthermore, since a confirmatory western blot test was not performed to reduce the number of possible non-specific false positive results, there is a potential for overestimation of the estimated prevalence of HSV-2. The discussed potential association between semen abnormality and HSV-2 should be also interpreted in light of the small sample size and potential involvement of other genital pathogens including, but not limited to, *Chlamydia trachomatis* (32–34) in semen abnormality.

Despite these acknowledged limitations, to our knowledge, this is the first study to screen the seroprevalence of HSV-2 IgG antibodies among patients seeking fertility treatment in the Abu Dhabi Emirate, UAE. The use of sensitive and specific ELISA kits provides potentially unbiased estimates on the observed prevalence of any time exposure to HSV-2 but not about the current infection with HSV-2. In the present study, collecting urethral and cervical swabs to perform molecular testing to investigate the prevalence of current infection with HSV-2 was also planned. However, none of the patients consented to provide such samples, and these samples were also not part of the routinely collected biological samples in the clinic. Our findings serve to address the gap in the literature on lifetime exposure to HSV-2 in specific population group and paves the way for future studies in the UAE.

## Conclusion

Exposure to HSV-2 at any time during an individual's life was not uncommon among patients seeking fertility treatment. More than one in ten patients were exposed to HSV-2 at some point in their lives. Having a higher prevalence of HSV-2 infection among males and male-induced infertility suggests a path for future studies to investigate the influence of HSV-2 on infertility. Proper health education campaigns to raise awareness of HSV-2 and reduce the risk of infection spread are also warranted to reduce risk transmission and sequelae of infection.

## Data availability statement

The raw data supporting the conclusions of this article can be made available upon reasonable request and approval by the authors. Requests to access the datasets should be directed to the corresponding author/s.

## Ethics statement

This study was reviewed and approved by Abu Dhabi Health Research and Technology Committee Institutional Review Board (DOH/CVDC/2020/1191) and HealthPlus Research Ethics Committee (REC/2020/P13). The patients/participants provided their written informed consent to participate in this study.

## Author contributions

NA and RA-R conceptualized and designed the study. NA, IA, LA, and SI contributed to the data collection. NA and JG performed laboratory testing. All authors have critically reviewed and approved the final manuscript.

## Funding

This study was funded by the Zayed Center for Health Sciences (ZCHS), College of Medicine and Health Sciences, United Arab Emirates University (Grant No. 31R181).

## Conflict of interest

The authors declare that the research was conducted in the absence of any commercial or financial relationships that could be construed as a potential conflict of interest.

## Publisher's note

All claims expressed in this article are solely those of the authors and do not necessarily represent those of their affiliated organizations, or those of the publisher, the editors and the reviewers. Any product that may be evaluated in this article, or claim that may be made by its manufacturer, is not guaranteed or endorsed by the publisher.
